# V. M. BEKHTEREV IN RUSSIAN CHILD SCIENCE, 1900S–1920S: “OBJECTIVE PSYCHOLOGY”/“REFLEXOLOGY” AS A SCIENTIFIC MOVEMENT

**DOI:** 10.1002/jhbs.21775

**Published:** 2016-02-22

**Authors:** ANDY BYFORD

## Abstract

In the early 20^th^ century the child population became a major focus of scientific, professional and public interest. This led to the crystallization of a dynamic field of child science, encompassing developmental and educational psychology, child psychiatry and special education, school hygiene and mental testing, juvenile criminology and the anthropology of childhood. This article discusses the role played in child science by the eminent Russian neurologist and psychiatrist Vladimir Mikhailovich Bekhterev. The latter's name is associated with a distinctive program for transforming the human sciences in general and psychology in particular that he in the 1900s labelled “objective psychology” and from the 1910s renamed “reflexology.” The article examines the equivocal place that Bekhterev's “objective psychology” and “reflexology” occupied in Russian/Soviet child science in the first three decades of the 20^th^ century. While Bekhterev's prominence in this field is beyond doubt, analysis shows that “objective psychology” and “reflexology” had much less success in mobilizing support within it than certain other movements in this arena (for example, “experimental pedagogy” in the pre‐revolutionary era); it also found it difficult to compete with the variety of rival programs that arose within Soviet “pedology” during the 1920s. However, this article also demonstrates that the study of child development played a pivotal role in Bekhterev's program for the transformation of the human sciences: it was especially important to his efforts to ground in empirical phenomena and in concrete research practices a new ontology of the psychological, which, the article argues, underpinned “objective psychology”/“reflexology” as a transformative scientific movement.

## Introduction

**Figure 1:**
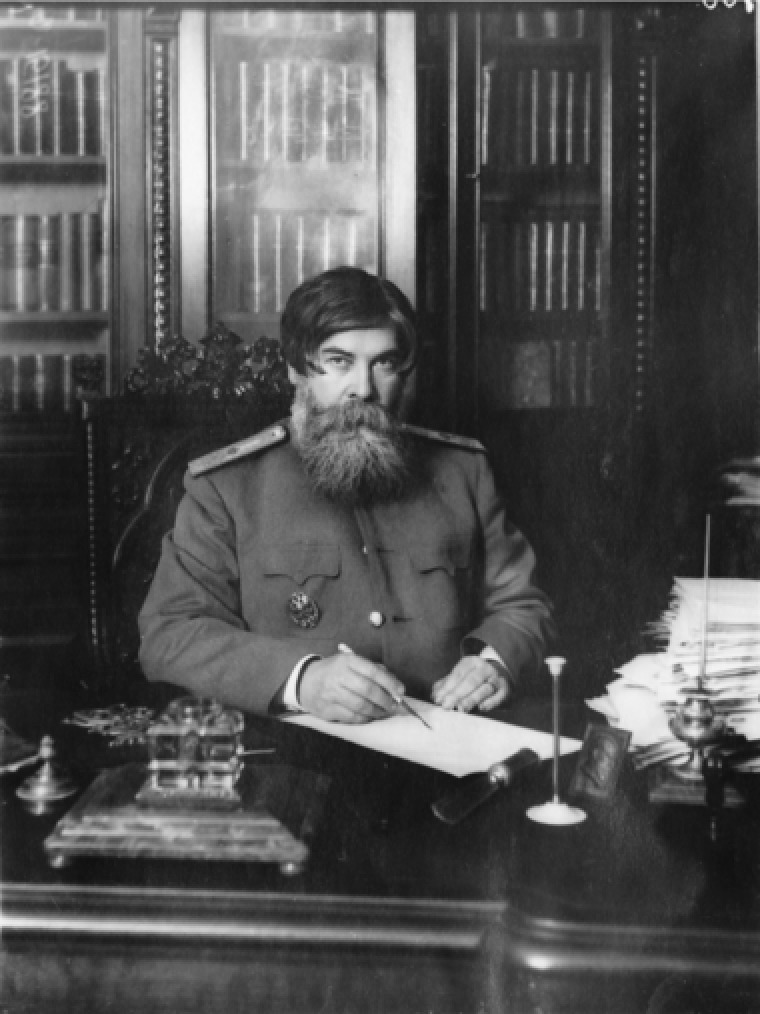
V. M. Bekhterev in his office at the Military Medical Academy in St. Petersburg (1913). Reproduced with the permission of the St. Petersburg Central State Archive of Cinematic and Photo Documents (TsGAKFFD, d. 15188)

Vladimir Mikhailovich Bekhterev (1857–1927) was one of the leading figures in the Russian human, medical, and behavioral sciences at the turn of the twentieth century. He was not only an authoritative and innovative neurologist and psychiatrist, but also a charismatic public figure, tireless organizer, and successful fundraiser, both before and after 1917 (Figure 1). An entrepreneur of science, he was responsible for establishing a wide network of research institutes and laboratories, clinics and higher educational establishments, journals and societies, helping raise within these an army of researchers, educators, and clinicians in a range of specialisms. History has, however, tended to keep Bekhterev in the shadow of his contemporary and arch rival, the Nobel laureate Ivan Petrovich Pavlov (1849–1936).^1^ Nonetheless, Bekhterev's role in Russia's early twentieth century psychology, psychiatry, and neurology is by now well recognized and documented,^2^ and this applies to his influences internationally, including the inspiration that some of his work has had on the pioneer of American behaviorism, John Watson.^3^

Bekhterev's name is associated especially with a program of research, which he in the 1900s dubbed “objective psychology,” and then, in the course of the 1910s, and especially after 1917, rebranded “reflexology.” There was some ambiguity about what these labels stood for—whether they referred to a particular theory and methodology, a research school led by Bekhterev, or even a distinct discipline. Bekhterev presented them in all these terms, but invariably articulated them as a program for overhauling the human sciences in general and psychology in particular. However, as entrepreneur and organizer, knowing that he needed to negotiate a range of interests and agendas, Bekhterev was, in practice, pragmatic enough not to insist dogmatically on programmatic uniformity. He advocated a multipronged approach to the study of human life, mobilizing a variety of perspectives and methodological strands into a collective endeavor, which he fostered under a more open notion of “psychoneurological sciences.”

Bekhterev's program for innovating the human sciences has to date been explored primarily in the context of the history of Russian psychology (Kozulin, 1984, pp. 49–61; Iaroshevskii, 1985, pp. 440–505; Joravsky, 1989, pp. 83–91, 273–276). Less attention has been paid to the place that “objective psychology” and “reflexology” occupied within “child science”^4^—the amorphous, yet dynamic field of scientific endeavor that flourished worldwide at the turn of the twentieth century, assembling a diversity of areas, including developmental and educational psychology, child psychiatry and special education, school hygiene and mental testing, juvenile criminology and the anthropology of childhood.

Bekhterev was involved—as researcher or facilitator—in most parts of the Russian child science movement, both before and after the October revolution. He is, in fact, often credited as one of its leaders (Balashov, 2012, p. 27). His role in setting up a host of institutions devoted to research into child development and pathology is invariably cited among his many achievements (Povarnin, 1926; Osipova, 1928b; Vygotsky, 1928). However, not enough attention has so far been paid to the importance that child development played within Bekhterev's program overall (Valsiner, 1988, p. 60). Nor has it been made clear what kind of a leader he was within Russian child science and what place “objective psychology” and “reflexology” occupied within it; or rather, how they related to contemporaneous rival programs focused on the study of child development.

This article will examine these very issues. It will foreground the strategic position that research into child development played in Bekhterev's program for the transformation of the human sciences in general and of psychology in particular. It will analyze the place that “objective psychology” and “reflexology” occupied within Russian child science in the 1900s–1920s. After explaining why in the late tsarist period “objective psychology” had rather less success in mobilizing support than certain other approaches (notably “experimental pedagogy”), it will follow the rise and fall of Bekhterevian “reflexology” in the course of the 1920s, focusing on its position in the state‐supported child science network which experienced extraordinary growth in the first decade of Soviet rule under the label “pedology.”

## Early Twentieth Century Child Science as Incubator of Scientific/Intellectual Movements (sims)

The early twentieth century was the period during which the child population became an important focus of scientific and professional interest, not just across Western Europe and North America (Depaepe, 1992; Boardman Smuts, 2006; Turmel, 2008), but also in other rapidly modernizing states, including late tsarist Russia and the early Soviet Union (Balashov, 2012). Scientific and professional work that claimed the developing child as a new object of research, expertise, and intervention was spread across a heterogeneous network belonging to a range of different, at that time still mostly emergent, disciplinary and occupational structures—above all those associated with medicine, psychology, and education. This field also depended on the active enrolment into science of nonspecialists, especially educated parents (as both amateurs and clients), on the patronage of philanthropists and civic organizations dedicated to public welfare, and on the support of political and bureaucratic structures, primarily those seeking to manage educational expansion and mass healthcare.

A number of participants in this growing field sought to turn it into a scientific discipline in its own right. When they did so, they sometimes used the neologism “pedology” (also “paedology” or “paidology,” from the Greek παῖς, παιδός, meaning “child”). They conceptualized it as a new specialization within the human sciences, devoted to establishing the biopsychosocial laws and norms of child development in view of determining rational principles of (individual and mass) childcare, upbringing, and education. The core innovation embedded in the idea of “pedology” was the conceptualization of the child as a distinctive object of scientific enquiry—an emergent and hence substantively different human form requiring new theoretical models and empirical methods of study.

Child science was a transnational phenomenon with many overlapping roots emerging simultaneously across Europe, North America, and beyond. In Russia, child science organizations started to mushroom from around 1900, although Russian interest in the “anthropological” study of child development as a function of upbringing and education dates back to the 1860s, most notably in the work of K. D. Ushinskii (1868–1869). By 1917, despite very limited support from the tsarist state, this field saw outstanding expansion among Russia's small, but growing and active, professional intelligentsia, especially those involved in the human sciences, education, and healthcare. After the October revolution, multidisciplinary research into children came under the wing of a rapidly centralizing socialist state and by the end of the 1920s, the Bolsheviks declared “pedology” an official science, entrusting it with the scientific management of child healthcare and education (Balashov, 2012).

In reality, however, child science—in Russia/USSR, as elsewhere—never crystallized into a coherent disciplinary formation, despite some concerted efforts to make it so, especially in the late‐1920s' drive to unify the field under a single, state‐led, and ideologically framed mission (Shvartsman & Kuznetsova, 1994). Child science remained a complex phenomenon, within which a host of different scientific technologies, research programs, and occupational structures arose. As a field that thrived on the idea of *innovation* in the human sciences, child science provided fertile ground for the emergence of a number of so‐called “SIMs,” in the sense given to this concept by Scott Frickel and Neil Gross (2005).

Blending insights from the social and historical studies of science, on the one hand, and the sociology of social movements, on the other, Frickel and Gross developed the idea of SIMs as a way of explaining how and why, under what conditions and to what effect, certain research groups and programs arise to alter (successfully or not) a preexisting scientific, intellectual, or professional landscape. According to their model, a SIM, as a framework for “innovation,” must have an explicit and (more or less) coherent program for scientific transformation (or “advance”), consciously devised as contentious relative to preexisting norms within a given scientific, intellectual, or professional realm. A SIM is inherently political, in the sense of being intent on the transfer of power within a given field. A SIM is ultimately constituted through organized collective action, through a process of enrolling participants and mobilizing resources. A SIM's program has to be circulated widely within the relevant community where it becomes subjected to scrutiny and contestation, and where it is embraced by some, while rejected by others. The success of a SIM is contingent upon the work done by the movement's participants to frame their program in ways that resonate with the concerns of the wider population inhabiting the field in question, often attracting those on the field's periphery or lower in the hierarchy. All of the above entails the development of particular rhetorical tactics. Crucially, however, a SIM is, by definition, an episodic phenomenon, an enterprise of transformation that, as such, exists only for a finite period and which, with time, invariably and inevitably transforms further, either disintegrating or shifting into some other form, within or outside of science.

To illustrate the above, I will briefly discuss the example of “experimental pedagogy,” which was arguably the most visible and successful SIM within Russian child science during the 1900s–1910s. Throughout this article, I will be using “experimental pedagogy” as a key comparator, useful for understanding the place and role of Bekhterev's “objective psychology” as a rival SIM within child science in this same period. Comparing the two SIMs’ overlapping yet distinct programs for scientific transformation, their differing target constituencies for wider mobilization, and their contrasting rhetorical tactics will show why the former was successful where the latter was not, and what was distinctive about Bekhterev's approach and ambition in child science.

Russian “experimental pedagogy” was led by the psychologist Aleksandr Petrovich Nechaev (1870–1948) and it thrived strategically on the boundary of psychology and pedagogy (Byford, 2008b). Inspiration for it came principally from Germany, especially the work of Ernst Meumann, but Nechaev developed it as a movement in his own original way, adapting it to Russian conditions (Romanov, 1997). “Experimental pedagogy” was particularly successful in positioning itself as a movement for transforming *pedagogy*, the latter being understood as the system of expert knowledge of the teaching profession, yet which at this time was not taught as a legitimate academic subject at Russian universities, since it was perceived as merely a normative synthesis of empirical practice, lacking scientific credentials (Byford 2008b).

“Experimental pedagogy” was promoted by Nechaev and his followers as a *new*, properly scientific approach to pedagogical matters, based primarily on the introduction of *experimental psychological research* (emblematic of a positivist human science) into education as an occupational field striving for full professionalization. The term “pedology” was also used by this group in the framing of their SIM, but invariably as a *de facto* synonym for “experimental pedagogy” as a new scientific basis for educational expertise rooted in experimental psychology (Byford, 2008b, pp. 73–79).

Not insignificantly, Nechaev's “experimental pedagogy” simultaneously sought to transform Russian *psychology*. At the turn of the twentieth century, Russian university psychology, based in philosophy departments, was slow to introduce experimental techniques on the model set by Wilhelm Wundt in Germany in the 1870s. Nechaev's SIM positioned itself as grounding Russian psychology in the natural scientific like “experiment.” His move included the introduction into Russian psychology not just of lab‐based research, but also, more controversially, mass experimental techniques, namely forms of mental testing (Byford, 2014).

## Bekhterev's “Objective Psychology” as a SIM

How did Bekhterev's “objective psychology” compare as a rival SIM operating in (roughly) this same arena at around the same time? Bekhterev was engaged in building a transformative scientific movement from the very start of his career in the mid‐1880s. His target of innovation were always, ambitiously, the human sciences in general, understood as the scientific framework for comprehensively explaining human life, including both its bioevolutionary underpinnings and its “higher,” psychosocial manifestations, individual as well as collective. While Bekhterev's background and institutional base were in medicine, his target of transformation was *psychology*. He identified psychology as occupying a pivotal position in the human and life sciences, situated at the juncture between the biophysiological and the sociohistorical. Psychology provided Bekhterev a strategically important access point to the domain of “the social,” which he was keen to capture from early on in his entrepreneurial career as a psychiatrist and neurologist (Bekhterev, 1928).

As mentioned, in late nineteenth century Russia, psychology's institutional home still lay in philosophy departments. Philosophy aspired to being the most generalist of disciplines, but struggled to legitimize its paradigms in the context of the proliferation of positivist sciences, the ranks of which psychology itself was starting to join following Wundt's establishment of experimental psychology. The significance of experimental psychology was certainly noted by Russian university philosophers in the last decades of the nineteenth century and was advocated by some (Grot, 1896), but with few attempts to emulate it in research practice (Budilova, 1961; Lomov, Budilova, & Kol'tsova, 1990). Bekhterev recognized that psychology in Russia remained vulnerable in terms of scientific legitimacy and that it was, consequently, left open to “innovation” from outside, above all from the vantage points of physiology and medicine (Budilova, 1961; Sirotkina, 2006).

In the late 1880s to early 1890s, as the newly appointed professor of nervous and psychiatric diseases at Kazan’ University, Bekhterev started to build his SIM on the basis of a tentative encroachment of “*neurology*” (understood as the anatomy and physiology of the brain and the nervous system) onto the established territory of psychology. Bekhterev's vehicles for this were his laboratory, as well as the new society of neurologists and psychiatrists and their journal, *The Neurology Herald* (*Nevrologicheskii vestnik*), that he established at Kazan’ University in 1893 (Brushlinskii & Kol'tsova, 1997).

In and of itself, this was hardly an original move, especially in the Russian context, where Bekhterev found direct inspiration in the ideas of the Russian pioneer of neurophysiology, Ivan Mikhailovich Sechenov (1829–1905). The latter had, in the 1860s–1870s, sparked a public debate over the question of “who was entitled to do psychology and how” (Sechenov, 2011). What was new in the 1880s–1890s, was that Bekhterev turned the arguments that in the 1860s–1870s were confined to an *ideological polemic* into the foundations of a transformative *scientific movement*—a program to be used to mobilize resources, institutional as well as human, in order to reshape psychology as a professional scientific endeavor, initially by claiming parts of it for a broadly conceptualized and expanding “psychoneurological” framework (Dupont, 2011).

What made Bekhterev's initiatives a SIM—a vehicle of transformation and innovation—was less his ambition to develop and extend the remits of “neurology” (as it was understood at the time), where he became internationally recognized as a major authority, but more his attempt to radically revise the established structures of “psychology.” Indeed, the key part of Bekhterev's campaign in the 1880s–1890s (like Sechenov's in the 1860s–1870s and Nechaev's in the late 1890s–1910s) was the critique of the current state of psychology. After he returned to St. Petersburg in the 1890s to assume the headship of the department of nervous and psychiatric diseases at his alma mater, the Military Medical Academy, Bekhterev's SIM started to crystallize around a new label—“objective psychology.”

“Objective psychology” has generally been viewed as part of the effort, typical of the late nineteenth century, to turn psychology into an exact science. This turn in psychology involved above all the introduction of experimental techniques inspired by and adapted from physics and physiology, in which the creation of laboratories was critical. Bekhterev's spell in Germany in the mid–1880s included his becoming acquainted with Wundt's seminar and laboratory in Leipzig (as was the case with Nechaev in the late 1890s). Bekhterev is, in fact, considered to be the founder of the first Russian laboratory to carry out research in experimental psychology along broadly Wundtian lines (Kol'tsova, 1985). He famously made his acceptance of the professorship at Kazan’ University in 1885 contingent on his being given the resources to set up a laboratory there (Brushlinskii & Kol'tsova, 1997). However, this laboratory operated principally in a medical context, as part of a neuropsychiatric clinic, and the experiments carried out there were mostly neuroanatomical and neurophysiological, and only secondarily experimental psychological. It was Nechaev, in fact, who claimed to have established Russia's first laboratory in experimental psychology properly speaking (in 1901, in St. Petersburg, at the Pedagogical Museum of the Department of Education of the Army Ministry), although the institutional context in which this laboratory operated was that of teacher training (Bol'shakova, 1990; Nechaev, 1990; Byford, 2008b, pp. 62–65).

While Bekhterev certainly emphasized experimentation as a major method of “objective psychology,” he did not, as Nechaev had done, turn “the experiment” itself into the bearer of innovation that defined his SIM. For Nechaev, whose background and immediate environment was psychology in philosophy departments and teacher‐training institutions, flagging “experimentation” on the model of the natural sciences was a radical and contentious move. Bekhterev's environment was, by contrast, medicine and neurophysiology, where emphasizing experimentation was hardly an innovation—his predecessors (e.g., I. M. Sechenov and S. P. Botkin) had already made this move in their studies of the nervous system (Kichigina, 2009).

Moreover, Bekhterev never restricted his program of “objective psychology” to the experimental method alone, but included in it, just as prominently, the method of “objective observation.” Here again, Bekhterev was hardly original. Not only was “observation” related to traditional clinical practice, but in the study of children, in particular, many participants developed systematic diary‐based programs for the observation of infants and preschoolers, often enrolling educated parents into these practices (Byford, 2013). Inspiration for it, in Russia as elsewhere, came especially from the work of Wilhelm Preyer, author of *Die Seele des Kindes* (Leipzig, 1881–1882), who developed and promoted the diary method from the 1880s.

Crucially, however, in contrast to other pioneers of Russian scientific psychology, Bekhterev was not satisfied with limiting his pursuit of “objectivity” strictly to reforming methodology, whether this involved (Wundt‐inspired) psychological experimentation or (Preyer‐inspired) objective observation. Practically all other innovators in Russian psychology and child science at this time sought objectivity in the *elimination of the subjectivism of the researcher*.^5^ In the case of experimentation this was to be ensured by delegating the recording and processing of data from the fallible humans to the impersonal laboratory apparatuses, test cards, and the statistical calculus (Byford, 2014). As for objective observation, the elimination of subjectivism was to be ensured by observer discipline, achieved through detailed instructions and training, carefully standardized observational rubrics, and by inciting the observer to continuous self‐critique (Byford, 2013, pp. 230–233).

To be sure, Bekhterev also followed and advocated these very same principles of methodological objectivity in the context of “objective psychology.” He emphasized the objectivity of laboratory experimentation and the registration of physiological data through apparatuses that he himself devised; he created his own distinctive system for the registration of observations that instilled in the observer the discipline of sticking strictly to recording external movements in relation to recordable stimuli while avoiding speculative mention of hypothetical internal states and motives.

However, Bekhterev's understanding of “objectivity,” *as defining of his SIM*, was not exhausted by the elimination of the subjectivism of the observer. What he simultaneously sought to eradicate was what might be called *the subjectivism of the observed*. Already in the 1890s, Bekhterev criticized Wundtian experimental psychology for its continued reliance on subjective, introspective, experiential data that, though restricted and controlled by the experimental framework, still needed to be communicated by the research subject for experimental measurements to acquire their full meaning and for the experiment to be complete (Brushlinskii & Kol'tsova, 1997).

Bekhterev grounded his “objective psychology” in the study of those categories where “introspection” (in Russian *samonabliudenie*, lit. “self‐observation”) was deemed to have *no* epistemological value. These were as follows: the mentally ill or the neurologically damaged (Bekhterev's stock in trade as a psychiatrist and neurologist); animals (whose “psychology” was beginning to be studied within an evolutionist framework, under the label “zoopsychology”); and finally, children—especially newborns, infants, and toddlers—whose distinct role in Bekhterev's program will be discussed in more detail in the next section.

Crucially, the innovation that lay at the core of Bektherev's “objective psychology,” and where he differed from his competitors in the same arena, was that the “objectivity” he was pursuing was never merely *epistemological*, but always also *ontological*.^6^ Bekhterev's ambition was to transform not just the way we know psychology, but the understanding of psychological reality itself. Bekhterev has often been accused of reducing “the psychological” either to “the physiological” (that one would study experimentally) or to “the behavioral” (that one would record through observation), or usually to both of these together. Moreover, it has been commonly argued that Bekhterev's reductionism remained ambivalent and inconsistent: while physiologists accused him of psychologizing physiological phenomena (such as the reflex, e.g.), psychologists criticized him for taking undue shortcuts between the biological and the social, via a fuzzy and shifting conceptualization of the psychological (Efrussi, 1923; Frankfurt, 1924; Kornilov, 1927; Protopopov, 1929).

It is generally recognized that Bekhterev's ontology was a monist one, in that he insisted on the physiological and the psychological (and in fact also the inorganic and the social) being part of a single, continuous reality, rooted in the same set of general laws (Bekhterev, 1904; Bekhterev, 1920–1922). The psychological was, for him, not reducible to the physiological; but nor was it merely an epiphenomenon; yet it could not be an autonomous domain, subject to its own laws, either (Bekhterev, 1997, p. 51). As I shall argue in the next section, *the developing child* (especially the infant and toddler) assumed a critical role in overcoming this paradox, and therefore also in Bekhterev's broader ambitions to transform psychology as a science.^7^

Bekhterev's other key criticism of what he called “subjective psychology” (which included Wundtian experimental psychology) was that its *language* remained misleading about the ontological nature of the phenomena it referred to. Bekhterev was scathing about the fact that this language still abounded in (in his words) “metaphysical” concepts, such as “attention” (*vnimanie*), “will” (*volia*), “memory” (*pamiat*’), “imagination” (*voobrazhenie*), and above all “consciousness” (*soznanie*; Bekhterev, 1997, p. 65). In his campaign to claim the territory of psychology, Bekhterev insisted on replacing this terminology with a new language that derived from the more advanced epistemology of “objective psychology” and that could then be put to work in the articulation of a new ontology of the psychological.

What Bekhterev was proposing was the remodeling of psychological realities into “reactions” to external stimuli. For example, what was traditionally called “attention” (*vnimanie*) was to be replaced by the “reaction of concentration” (*reaktsiia sosredotocheniia*; Vladychko, 1909). Bekhterev envisaged this language as coming directly out of his methodological setup. The term “reaction of concentration” acquired meaning as an inherent part of particular ways in which the phenomenon of “attention” (a classical topic of experimental psychological research) was observed, recorded, measured, and analyzed in Bekhterev's laboratories (Povarnin, 1906).

However, Bekhterev and his followers regularly accompanied their innovative “objective” terminology with the more commonplace “subjective” one. The use of the term “reaction of concentration” invariably prompted a rhetorical gloss explaining that this term referred to what “old” psychology used to call “attention.” The translation from one terminology to the other also relied on intermediary formulations: for example, the explanation that the “reaction of concentration” meant “roughly, […] an organism's orientation (*ustanovka*) towards attentive activity (*vnimatel'naia deiatel'nost*’)” (Artemov, 1929, p. 50). What this amounted to was not the replacement of one set of signifiers with another, but the possibility of shuttling from “old” to “new” language, as required by the rhetorical context.

One must not underestimate the tactical value of this translation between “old” and “new” psychology. The transformation of psychology's language was part of the innovation that was being introduced: the new language served as both a tool and an effect of the proposed scientific advance. Bekhterev's claim was, of course, that the new terminology was more “scientific.” However, it was also less familiar, its esoteric character preventing its acceptance by a broader constituency of supporters, especially among parents, teachers, and doctors. Yet, the mobilization of non‐ or semispecialists remained vital to any SIM that sought to reform psychology, given that the discipline was at this point still professionally undeveloped, demanding substantial engagement and participation from the wider educated public and other, contiguous professional groups.

The effectiveness of the rhetorical tactics of Bektherev's “objective psychology” can be usefully contrasted with the very different rhetorical tactics of Nechaev's “experimental pedagogy.” Nechaev preserved all the familiar notions of (in Bekhterev's terms) “subjective psychology,” but turned a single term—“*experiment*”—into the hallmark of “scientificity” that his SIM was bringing to the fields he was seeking to transform—pedagogy and psychology. That tactic, because of its simplicity, proved (for a while at least) rather effective in mobilizing both specialist and nonspecialist support, although it was by no means uncontroversial, meeting with stiff opposition and criticism from university‐based psychologists as well as some more conservative teachers (Byford, 2008a, 2008b, 2014, pp. 26–35). By contrast, Bekhterev's SIM relied on the rhetorically far more laborious process of continuous translation back and forth from the unfamiliar language of “objective psychology” to the better established and more commonplace terminology of “subjective psychology.” This proved much less effective in mobilizing wider support, especially among teachers.

## The Child and The Reflex: Bekhterev's Position in Late‐Tsarist Child Science

Another key difference between “objective psychology” and “experimental pedagogy” is evident in the child population that Bekhterev's program focused on. While Nechaev targeted primarily children of school age and emphasized the educational context, Bekhterev prioritized infants and toddlers. This interest in the earliest phases of development is traceable to Bekhterev's original, 1880s’ research, which was concerned with the development of the nervous system based on neuroanatomical preparations of the brains of embryos and newborns. Bekhterev made the most of the “embryological method,” which he learned from its pioneer, Paul Emil Flechsig, during his 1884 stay in Leipzig (publishing the results in his highly regarded *The Conducting Paths of the Spinal Cord and the Brain*, 1896–1898).

Bekhterev's keen interest in child development *per se* began in the 1900s, in the context of the more general expansion of Russia's child study movement, in which he did not fail to spot opportunities for entrepreneurial action, especially for expanding and promoting “objective psychology.” Emulating the European classics of child study, Bekhterev began with observations of his own children, especially his youngest daughter Mariia, born in 1904, bringing on board, as a fellow observer, his wife Natal'ia Petrovna (Bekhterev, 1910, 1912; Nikiforov, Amirov, & Mukhamedzianov, 2007, p. 110).

From 1907, Bekhterev's most significant contributions to child science were tied to the work of the so‐called Pedology Institute (Pedologicheskii Institut; hereafter PI), which he established as a small, but important, part of his grand project at that time—the Psychoneurological Institute (hereafter PNI).^8^ This was also the period during which Bekhterev wrote and published his *Objective Psychology* (1907–1910). The term “pedology” in the title of the above institute conceptualized the latter *not* as a “new pedagogy” (as Nechaev and his followers were doing at that same time), but as one of many structurally necessary parts of an expanding grand edifice of the “psychoneurological sciences,” to which PNI as a whole was devoted. The difference between the two visions of “pedology” was evident during discussions at the first congress in pedagogical psychology in St Petersburg in 1906. Nechaev proposed the formation of a Pedology Institute that would be devoted to the “pedological scientific foundations of pedagogy”; Bekhterev was critical of this, seeing a Pedology Institute making sense only as a subsidiary part of a broader structure, namely his idea for the PNI (Balashov, 2012, p. 54).

In reality, PI was a rather modest enterprise, built on relatively limited private donations and initially housed in a flat,^9^ although it acquired its own premises on PNI's site in 1913.^10^ The Institute functioned essentially as a lab‐nursery. A handful of children (initially rather sickly ones in need of care) were brought there from a very young age and were looked after by staff for several years, six or seven in some cases.^11^ The children's development and behavior were meticulously observed, recorded, and experimented on, mostly by a resident female psychologist (*ordinator*), who usually had medical training, while a female educator (*vospitatel'nitsa*) with specialist pedagogical training and experience was responsible for their daily care and upbringing (Psikho‐Pedologicheskii Institut, 1908; Kostin, 1910; PI, 1912). A resident pediatrician monitored the children's health.^12^ Bekhterev and others formed a committee that oversaw the work of the establishment and the research carried out there. From 1910, PI came under the headship of Bekhterev's former student, Konstantin Innokent'evich Povarnin (1877–1963), who organized the work of the Institute in a more systematic way (Povarnin, 1919; Skhor, 2002), including its contribution to PNI's teaching program.^13^

Diaries, three separate kinds—physiological, psychological, and pedagogical—were kept for each child, following standardized rubrics. The physiological diary contained anthropometric measurements and recorded bodily temperature, pulse rate, respiration and sleep patterns, appetite, mood, and illnesses. The psychological diary was based on registrations of reactions to external stimuli, mostly grimaces and movements. Photography was also used.^14^ The focus of research was on the development of sensory organs and motor skills, on shifts from instinctive and reflexive reactions to cognitive (spontaneously exploratory, as well as conditioned or learned) behavior, and on the evolution of what was conceptualized as “symbolic” behaviors, including speech, drawing, craft, and play.^15^

**Figure 2:**
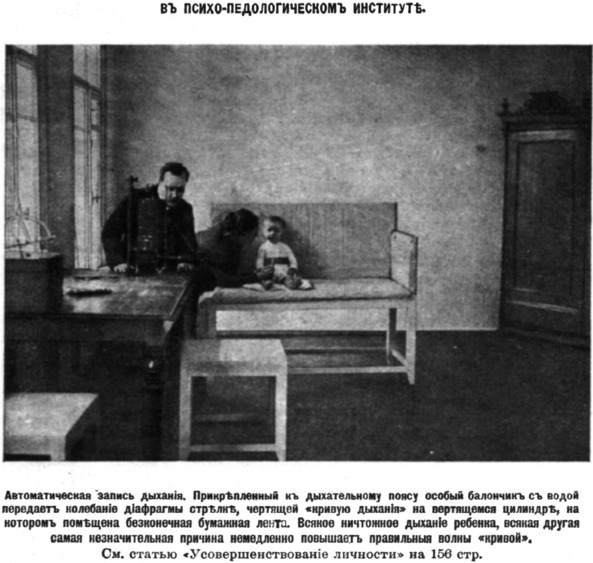
At the Psycho‐pedological Institute. “The automatic registration of breathing. Attached to the breathing strap is a special balloon filled with water, which conveys the vibrations of the child's diaphragm to a needle that charts a ‘breathing curve’ on a rotating cylinder with an endless paper ribbon. Even the lightest disturbance of the child's breathing for whatever reason immediately raises the regular amplitude of the ‘curve.’” From the article “The Perfecting of Personality,” published in the newspaper *New Time* on April 2, 1908 (Usovershenstvovanie lichnosti, 1908).

Observations were combined with experiments, and these were often carried out by visiting researchers, mostly doctors studying for a higher degree. There was particular interest, for instance, in measuring changes in respiration under specific stimuli, for example, auditory ones. This methodology was deployed in studying the “reaction of concentration” (i.e., “attention”) by means of recording interruptions in the child's breathing pattern upon, say, hearing a sound (Figure 2). Such research was performed with the help of apparatuses designed by Bekhterev himself, which measured and graphically displayed fluctuations in breathing, producing pneumograms as objective visualizations of the reaction in question.

**Figure 3:**
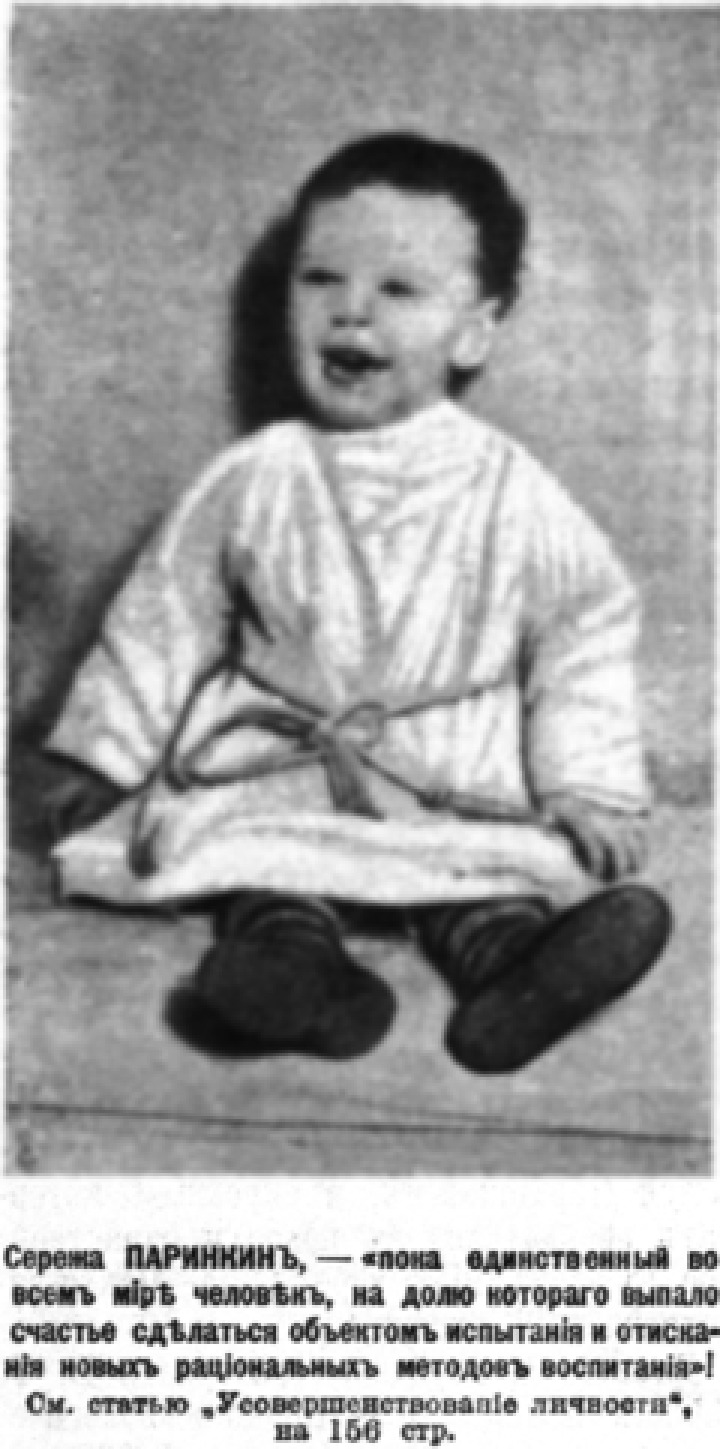
Serezha Parinkin. “So far the only individual in the world lucky enough to become the object of experimentation in the search for new rational methods of upbringing!” From the article “The Perfecting of Personality,” published in the newspaper *New Time* on April 2, 1908 (Usovershenstvovanie lichnosti, 1908).

Finally, there was the pedagogical diary that registered how particular forms of educative stimulation affected the child's behavior in particular conditions. The fact that PI depended on private sponsorship meant that Bekhterev could not afford to be too narrow in defining the purpose of his enterprise. He had to remain eclectic in his promotional strategy, pursuing every angle that was likely to yield donations. He highlighted the innovation that science would bring to education, just as Nechaev was doing (Arkatov, 1908); he piggy‐backed on the widespread circulation in the Russian media of eugenic concerns with “degeneration” and utopian promises of scientifically engineered human perfection (Usovershenstvovanie, 1908; Figure 3); he emphasized the patriotic duty of Russians to develop their own scientifically grounded ways of rearing their young, especially in competition with the Germans (Bekhterev, 1909b); and he relied on the interest and investment of Russia's educated classes in problems of “upbringing” (*vospitanie*; Bekhterev, 1908b), positioning himself even as something of a childcare guru (Bekhterev, 1909b; 1911; 1913).

In this context, the supposedly new, scientifically grounded childcare that took place at PI was in reality mostly an adaptation of middle‐class home rearing with elements of the progressive educational experimentation that was becoming popular in some of the private kindergartens. Bekhterev invoked the popular Froebel kindergartens as a major competitor, which his scientific approach to early upbringing was expected to outdo (Bekhterev, 1909). Indeed, the science that went on at PI was also intended to inform Russia's budding (privately run) system of preschool education (TsGIA f. 2265 op. 1 d. 929 ll. 4–6ob). Research carried out at PI was promoted especially to the Society for Preschool Education and at the First All‐Russian Congress on Family Education in 1913 (Gerver, 1914; Trudy, 1914, pp. 266–349).

The emphasis that Bekhterev placed on the study of infants and toddlers was distinctive within Russian child science of the 1900s, where other leading scientific players focused rather more on children of school age or late preschoolers at the youngest. Observations of the earliest phases of child development tended to be the domain of *parent‐amateurs*, not least mothers, who associated this practice with the tasks and problems of home rearing (Byford, 2013). Bekhterev's own choice of this object of study was determined by his underlying research concerns: he construed infants and toddlers as *models* for understanding the genesis of “neuro‐psychic activity.” He presented this as the root problem of psychology—namely, the question of how higher mental functioning and complex behaviors emerge out of and in contiguity with physiological processes, in environmental conditions typical of human development (Bekhterev, 1912). Bekhterev stressed the importance of the earliest stages of psychological development for the subsequent formation of adult personality (especially as the potential source of psychopathology, an area where he spoke with authority as a psychiatrist). However, even more important was the supposed psychoneurological *simplicity* that the child's organism presented to the researcher—the seemingly elementary character of behavioral and physiological changes that one could observe and measure in a laboratory setting. And yet, as I shall go on to explain, Bekhterev's focus on such young children was also one major reason for the fact that, irrespective of his authority and prominence, “objective psychology” was less successful a SIM within child science than Nechaev's “experimental pedagogy.”

According to Frickel and Gross, a SIM is most likely to be successful when led by an established, high‐status intellectual actor, yet who harbors complaints against what s/he understands to be the prevailing intellectual tendencies of the day. In this respect, Bekhterev was a model SIM leader: while he was even more radical than Nechaev in his critique of the existing paradigms of psychology, he was also unquestionably more authoritative and publicly better known. Nechaev was trained at St. Petersburg University's philosophy department, but after disagreements with his supervisor, A. I. Vvedenskii, he failed his master's defense and was barred from a university career (Lomov, Budilova, & Kol'tsova, 1990, pp. 200–213). He was thus forced to build his base for “experimental pedagogy” in newly emerging teacher‐training institutions, on the peripheries of Russia's academic network. By contrast, Bekhterev, after training at St Petersburg's Military Medical Academy, was first Head of the Department of Nervous and Psychiatric Diseases at Kazan’ University (1885–1893) and then led the equivalent department and clinic back at the Military Medical Academy (1893–1913). He even became the Head of this institution in 1905, but then clashed with the tsarist authorities on political grounds in the early 1910s and was sacked in 1913. At the same time, in the course of the 1900s–1910s, Bekhterev was—like Nechaev, and often in collaboration with him—actively engaged in building consciously innovative research centers and higher educational establishments, of which PNI was the prime example.

Bekhterev thus quickly became one of the most respected figures in late tsarist Russia's flourishing child science movement. He served regularly as presiding chair at Russia's most significant conferences in this field (although it was Nechaev who was usually the principal organizer, which is why these conferences were first called conferences in “pedagogical psychology” and then “experimental pedagogy”; cf. Sokolov, 1956a, 1956b). Bekhterev's PNI and the journal *Herald of Psychology, Criminal Anthropology and Pedology* that he edited were among the core institutional bases for Russian child science of this era.^16^ These fostered research that was by no means restricted to Bekhterev's own program.^17^ It was also Bekhterev who represented Russia at the First International Pedology Congress in Brussels in 1911.^18^

*And yet*, despite Bekhterev's personal authority and entrepreneurial activism, “objective psychology” did not achieve prominence as a SIM within late‐tsarist child science. In contrast to Nechaev's “experimental pedagogy”—a movement which strategically targeted the population of professionally highly interested and organized *teachers*—Bekhterev's program lacked a similar professional constituency for broader mobilization. Given his focus on infants and toddlers, Bekhterev's primary audience could only be educated parents (who, even when actively engaged and organized, remained a diffuse amateur group) and young female staff working in tsarist Russia's relatively few preschool institutions, who were in occupational terms still poorly professionalized. When Bekhterev tried to appeal to schoolteachers (e.g., at the conference in experimental pedagogy in 1916), he, at cross‐examination, had to concede that his methodology's contributions to teaching practice remained a matter of future research (Bekhterev, 1916).

The other reason for the limited role that “objective psychology” played in late‐tsarist child science was that Bekhterev never restricted the remit of his SIM, as a program for innovation, to the realm of child science itself. Nechaev had been forced into this move by the fact that, while starting off in university psychology, he had to develop his institutional base and program for innovation within teacher training. For Bekhterev, by contrast, child science was a specialist subdomain of a much broader, complexly integrated structure of the human and behavioral sciences that he was ambitiously building (Bekhterev, 1908a). This was why within the field of child science Bekhterev refrained from an overly partisan role, which Nechaev had strategically adopted in his promotion of experimental psychology to teachers. Bekhterev instead chose the role of higher authority and neutral arbiter. What is more, he preferred to delegate actual research in child science to others—primarily staff at PI.^19^

Nevertheless, even if the field of child science as such was not Bekhterev's prime target of innovation, child development occupied a crucial role in the articulation of the theory that lay at the core of his program for transforming psychology as a science. Child development had, in fact, been vital already to Sechenov's revision of psychology's premises in the late nineteenth century. Sechenov devoted much of the third and final chapter of his “Who Should be Doing Psychology and How?” (1873) to an elaboration of how in early childhood “psychology” emerges as a set of reflexes, out of which, gradually, elements of thought and willful action arise. In the opening line of this chapter, Sechenov effectively *identified* the developing infant with the reflex: “In infancy and childhood all psychic phenomena have the character of reflexes” (Sechenov, 2011, p. 164; translation mine). Sechenov used the description of the reflexes of the developing child as proof of the continuity between physiological phenomena and higher mental functions. Bekhterev developed this idea further within his own research setup; the work carried out at PI was crucial to this.

The various techniques of laboratory experimentation and objective observation that Bekhterev and his followers combined at PI can be understood as research assemblages operating on the boundary between the “physiological” and the “psychological.” In this context, “the child,” systematically observed and experimented upon, was turned into an instrumental component of this research assemblage—*a registration device in its own right*. In Bekhterev's understanding, the child was *neither* a “research subject” (as Wundtian experimental psychology or Nechaev's experimental pedagogy might understand it); *nor* was the child an “object of study” (as pedology, the “science of the child” *stricto sensu*, might claim it). Instead, the child (or rather, the earliest human developmental forms—embryos, newborns, infants, and toddlers), whose movements and reactions, gestures and grimaces, pulse rates and breathing patterns were meticulously recorded by researchers and measured by apparatuses, served as a *black box* at the heart of said research assemblage.

The purpose of this assemblage was to model *translations* on the boundary between the biophysiological and the psychosocial (Bekhterev, 1997, pp. 72–82, 88–92). The principal purpose of the “black box” that the developing child embodied in research practice and figured in psycho‐developmental theory was *to conceal* the work of translation on this boundary. “The child,” on the one hand, *encapsulated* and, on the other, *screened* transmutations between and across “the physical” and “the mental.”

Decisive to this operation was the creation of a particular *schema of translation*, to be understood as a kind of key, or rather, an internal mechanism of transformation that one needed in order to perform the operation of translation on this boundary. Bekhterev identified this “schema” in *the reflex*. The reflex was the elementary mechanism of the central nervous system, as established by Sechenov in the 1860s and then expanded upon by Pavlov in his theory of conditional reflexes in the early 1900s. Contemporaneously with Pavlov, Bekhterev developed his own, related theory of secondary reflexes, branding them “associational” (*sochetatel'nye refleksy*) rather than “conditional” (*uslovnye refleksy*).^20^ Crucially, though, “the associational reflex” was here not so much a phenomenon of the nervous system, but a transferrable model, or in Bekhterev's terminology a “schema” (Bekhterev, 1997, pp. 54, 88–92), that could shift from sensorimotor physiology to (conscious and unconscious) psychology, and then to individual and social behavior (Dupont, 2011).

The idea behind Bekhterev's “associational reflex” *as a schema*, was that simple, inherited reflexes (or reflexes proper, with which an organism was born) served as kernels for the gradual building of secondary reflexes created developmentally in the central nervous system through the organism's continuous exposure to stimuli in the environment. The sum of associational reflexes ultimately accounted for fully developed human behavior, including psychic life, which ought, therefore, at least in principle, to be describable in terms of elaborate complexes of associational reflexes. While it was vital to Bekhterev that “the reflex” should remain a neurological reality (thus linking psychic life to nervous activity), his theory of associational reflexes worked not as a description of a physiological mechanism (which was the principal purpose of Pavlov's conditional reflexes) but as a means of accounting for the full complexity of an organism's relationship to the external and internal environment (Bekhterev, 1997, p. 56).^21^

Furthermore, “the associational reflex” was constructed as essentially a *developmental model*. It was a mechanism through which a black box (embodied by “the child”) progressed, cumulatively, from a starting position, where all inputs (i.e., stimuli) led to only a handful of highly predictable, if diffuse, outputs (involuntary movements or some physiological reaction in a very young child), to a position where the outputs became far more precise, but at the same time, in their totality, highly complex (and hence far less predictable or controllable) ranges of possibilities (with which adult human behavior and higher psychological functions were to be identified).

## BEKHTEREV IN EARLY‐SOVIET CHILD SCIENCE: THE RISE AND FALL OF “PEDAGOGICAL REFLEXOLOGY”

The abstract, and hence highly transportable, schema of “the reflex” became so important to Bekhterev that, from the late 1910s, and especially after 1917, he converted the title of his program from “objective psychology” to “reflexology.” This new program was launched through his books *The General Foundations of Reflexology* (1918), *Collective Reflexology* (1921), and *The General Foundations of Human Reflexology* (1923). The reason for this rebranding is more complex, of course, and involved Bekhterev's broader repositioning of his program in the rapidly transforming ideological, epistemological, and institutional arena of the human sciences under the Soviet regime (Joravsky, 1989, pp. 273–276).

In the early Soviet Union, it was expected that the epistemology of psychology would be “revolutionized.” Bekhterev now had to contend with a host of new players stepping onto his territory, including not just Pavlov, but also the new director of the Moscow Institute of Psychology, K. N. Kornilov, who was turning psychology into (Marxist‐framed) “reactology,” and P. P. Blonskii who later became a leading Soviet pedologist, but who was at the start of the 1920s mostly promoting a version of American‐inspired behaviorism (Efrussi, 1923). The remainder of this article will focus on the place that Bekhterev's “reflexology” occupied specifically in early‐Soviet child science.

The 1920s saw a remarkable expansion of the network of child science institutions and endeavors in the Soviet Union (Balashov, 2012). Research that focused on children greatly benefited from, but also became strongly dependent upon, centralized state sources of funding and recognition. The Bolsheviks invested keenly in child science, seeing it as one of a number of exciting new strands of the human sciences that could be harnessed for the purposes of accelerated modernization and revolutionary social engineering (Kurek, 2004). Child science fed especially into education and healthcare, promising to develop new techniques of population management, eugenic transformation, and social emancipation.

The consequence of state support for child science was that, while the expanding network maintained its disciplinary and occupational heterogeneity, the different groups and actors rallied increasingly under a single label—namely “pedology”—which came to be recognized by the new patrons (the Soviet state) as the preferred designation for the totality of scientific research focused on the child population. However, despite the rhetorical support that grew around “pedology” (including its reification and instrumentalization as a scientific discipline), for most of the 1920s it functioned essentially as a “*bandwagon,*” rather than a disciplinary structure.^22^ “Pedology” was an officially supported (i.e., legitimate) *framework of convenience*, within which a diversity of research programs mushroomed and thrived.^23^ Certain regularities of practice, standardizations of methodology, and fixations of core objects of interest were certainly being established (Shvartsman & Kuzentsova, 1994). Nonetheless, *in research practice*, Soviet “pedology” remained fragmented across a wide network of different (and continuously restructuring) institutions and methodologies. There was, moreover, constant jostling for position, with little overall consensus and no single research program dominating to the exclusion of others. The different groupings treated each other as both allies and rivals. They were driven to collaboration and support when campaigning for state investment in the overall enterprise. But when it came to developing specific approaches, their interaction was usually animated by bitter divisions and mutual criticisms. From time to time, however, certain programs and agendas did acquire greater prominence and influence, at least temporarily and within specific contexts.

Bekhterev operated just as successfully under Bolshevism as he had done under tsarism, continuing with his remarkable scientific entrepreneurialism in the radically changed circumstances of the 1920s. Immediately after the October revolution, Bekhterev's principal home institution, the PNI, experienced major restructurings, both as an educational establishment and as a research cluster, with PI being taken out of it. The Bolsheviks initially allocated the financing of PI to the bureaucracy responsible for preschool education, but the latter considered it too expensive to run even as a “model nursery” and closed it down in 1919 (TsGIA f. 2265 op. 1 d. 930 l. 1; TsGIA f. 2265 op. 1 d. 932 ll. 1–17).

In 1922, Bekhterev engineered the creation of a new Pedology Institute, to be financed from the state science budget. This new structure had a broader remit and was not restricted to Bekhterev's own reflexological agenda: its leading departments were, in fact, experimental pedagogy, which focused on schoolchildren, and observational psychology, which focused on preschoolers, although there was also a small department in “developmental reflexology” (*geneticheskaia refleksologiia*; lit. “genetic reflexology”), run by another follower of Bekhterev, N. M. Shchelovanov (Shchelovanov, 1929; Martsinkovskaia, 1990). This new PI disassembled further in 1925, its different departments being reallocated to different institutions. From 1918 Bekhterev was simultaneously building a larger research base for “reflexology” more generally—the Reflexological Institute for the Study of the Brain (or Brain Institute for short), which included the study of child development (Bekhterev & Shchelovanov, 1919; Shchelovanov, 1921). Shchelovanov's department in “developmental reflexology,” which was effectively continuing the research carried out at the original, prerevolutionary, PI, moved there fully in 1922 (Balashov, 2012, p. 74).

It was clear to Bekhterev that his program's contributions to child science had to respond to the urgent demands of the new state, given that the Bolsheviks were calling upon loyal experts, such as himself, to help modernize education and healthcare. In the early 1920s, the priority became the “difficult” and “defective” children—the millions of orphans and waifs (the so‐called *besprizorniki*, lit. “the unsupervised”)—a problem unprecedented in scale and one of the most important foci of Soviet state intervention in the wake of the revolutionary civil war (Ball, 1994). As part of what was known as the Psychoneurological Academy (PNA), a structure that developed out of the earlier PNI, Bekhterev set up a host of new institutes concerned with developmental pathologies, juvenile delinquency, and special education. These included the Pedagogical Institute for the Socialization of the Normal and the Defective Child, the Child Diagnostic Institute (Detskii obsledovatel'skii institut; DOBI), the Educational‐Clinical Institute, and the Institute of Moral Education (Balashov, 2012, pp. 73–78).

In the mid‐1920s, however, it was the Bolshevik campaign to expand education and introduce radical school reforms that drove the growth of the Soviet child science network. These developments coincided with the Second All‐Union Psycho‐Neurological Congress in January 1924, in Petrograd, which Bekhterev used to further the cause of “reflexology” as the leading SIM in this field. This event attracted teachers as well and encouraged some of Bekhterev's followers to promote his program more actively as a foundational framework for a new educational science, both in terms of providing a reflexologically informed understanding of the education process and in terms of developing efficient (reflex‐based) techniques for forging new skills or undoing bad habits.

This development was connected to the Taylorist‐inspired move by the Soviet Commissariat of Education to model Soviet education on the principles of the scientific organization of labor (*nauchnaia organizatsiia truda* or NOT). Bekhterev's followers strategically extended their reflexological research in the direction of the school‐age population, focusing especially on aspects of the education process that correlated with industry.^24^ They studied the pupils’ relationship to work, levels of concentration, speed and accuracy of attending to particular school tasks, stamina, and exhaustion patterns. This research was developed primarily within so‐called “pedagogical reflexology” (*pedagogicheskaia refleksologiia*), the base for which was the laboratory at the Brain Institute run by Vera Nikolaevna Osipova (1876–1954).^25^ The underlying ambition of “pedagogical reflexology” was to base teaching on the organization of class activities as a system of stimuli for generating and fixing desired responses (Protopopov, 1928, pp. 6–7).

The primary methodology used by Osipova was experimentation, above all the artificial creation of secondary reflexes. The experiments that Osipova carried out on schoolchildren grew out of the experiments that Bekhterev had devised at his psychiatric clinic in the 1900s. These were arguably the closest to Pavlov's experiments on dogs, although they had as their output physical movement, rather than salivation, and they used as stimulus electroshocks, rather than food. Osipova's experiments involved placing the child in an isolated chamber and then artificially creating “associational reflexes” by applying a low electrical current to the child's hand “in association” with other forms of visual and/or audio stimuli.^26^ In somewhat younger children, or children who displayed anxiety during the experiment, electroshocks were replaced by verbal commands. The latter was subsequently presented as a methodological development likely to be of practical use in the classroom (Osipova, 1928b)!

Those who saw themselves as followers of Pavlov rather than Bekhterev presented their own fusion of reflexology and pedagogy in not dissimilar terms. The key example here is I. A. Ariamov who taught reflexology at a number of teacher‐training colleges and published a series of textbooks, closely aligning reflexology to educational reforms that were at the time being implemented by the Commissariat of Education, namely the importation from the United States of what was at the time known as the Dalton Plan (Ariamov, 1925, 1926a, 1926b, 1927b; Ivanova, 1928). While the reflexologies of Bekhterev and Pavlov need to be distinguished, the “reflexological” model of educational research advertised to teachers was fairly uniform, regardless of whether it referred to Bekhterevian associational or Pavlovian conditional reflexes.^27^

As Raymond Bauer (1952, pp. 55–57) has argued, Bekhterev's and Pavlov's reflexology intermixed in the popular mind of this era, creating some confusion, but also helping increase prestige. In the mid‐1920s, the Ukrainian Commissariat of Education briefly decreed that “reflexology” rather than psychology was to serve as the basis for scientific pedagogy. The leading figure in the Bolshevik educational establishment at the time, A. P. Pinkevich, spoke positively about reflexology's promises for education. Just as was the case with “objective psychology” earlier on, the promotion of “reflexology” to a wider constituency, especially the education profession, demanded forms of translation (in both directions) between the esoteric language of “reflexology” and the language of traditional pedagogical frameworks and lay psychological concepts familiar to teachers. This translational work was carried out by intermediaries, such as Ariamov (1927b), who published articles on “pedagogical reflexology” in key educationalist journals.

However, just as had been the case in the tsarist era, this did not prove particularly effective. The education profession remained unconvinced, not least because “reflexology” (whether Bekhterevian or Pavlovian) was a relative latecomer to the field of educational research. “Reflexology” was here stepping onto a territory simultaneously claimed by a number of other innovative child science programs promoted by groups led by figures such as Pavel Blonskii or Lev Vygotskii who were closer both to the education profession and to the educational administration. What is more, the latter were getting increasingly impatient for speedy results, while work on conditional or associational reflexes was invariably accompanied by caveats that more time was needed before such foundational research would lead to techniques that could be applied in the classroom.

On the eve of the First All‐Union Pedology Congress in Moscow (27 December 1927 to 4 January 1928), the purpose of which was the state‐directed “integration” of the pedology “bandwagon” in preparation for the forthcoming first five‐year plan (Griboedov, 1929), Bekhterev passed away in mysterious circumstances, transforming the atmosphere of the event into one of mourning. While the obituaries published at the time followed the *de rigueur* high praise of the deceased's contributions, his untimely death no‐doubt sped up the demise of “reflexology” in subsequent years. It could be argued, however, that already by the time of this congress, “reflexology” had been reduced to a specialist, and increasingly marginal, approach within the broader field of Soviet child science. Indeed, the most that Osipova could do at this event was position her “method of associational reflexes” alongside other “methods,” especially mental testing ones (Osipova, 1928a; Osipova 1929). Pavlovian “pedagogical reflexology” promoted by Ariamov was also in decline, his approach being forced to melt into a loose pedological synthesis (Ariamov, 1927a). Shchelovanov's “developmental reflexology” was reduced to a niche area under the remit of the Commissariat of Health (Schelovanov, Figurin, & Denisova, 1928).

Stalin's Great Break (1928–1929) brought about a turbulent phase of political realignment and institutional restructuring, sackings, and purges that affected most strands of Soviet science, not least the “psychoneurological” ones. Practically *all* movements and programs within it, not excluding “reflexology,” came under ideological attack and were subjected to vitriolic denunciations and political disciplining (Umrikhin, 1991). Bekhterev's followers working at the Brain Institute tried to rescue the enterprise (Kurazov, 1928; Kurazov 1929; Anan'ev, 1929; Refleksologiia ili psikhologiia, 1929; Refleksologiia i smezhnye napravleniia, 1930), especially through extensive ideological and methodological “self‐critique” (*samokritika*), which included public declarations of commitment to purge their research of “Bekhterevism” (*bekhterevshchina*).

During the 1930s, Bekhterevian reflexology was first subjected to revisionism (Osipov & Shirman, 1932) and then faded out of the Brain Institute altogether (Umrikhin, 1991). Researchers shifted their work in the direction of a more Pavlovian‐like, physiological approach. Shchelovanov's research on infants, for instance, limited itself to the study of sensory reception and motor activity (Osipov, 1938; Anan'ev, 1939). What is more, this work had to be completely disassociated from child science as such, since “pedology” suffered an even more catastrophic demise at this juncture: in July 1936 the Communist Party denounced it as a pernicious “pseudoscience” in the context of Stalin's campaign to bring into line the Commissariat of Education, which he perceived as the bastion of the old Leninist guard (Kurek, 2004).^28^

## Conclusion

In early twentieth century Russia, the expanding field of child science became a vibrant arena in which a large number of research programs and scientific agendas arose and competed, mobilizing support among the wider educated public, occupational groups, civic organizations, and parts of the state apparatus with vested interests in this area. One of the key players in this field was the neurologist and psychiatrist Vladimir Mikhailovich Bekhterev. As an active participant in the expansion of Russia's network of child science institutions in the 1900s–1920s, Bekhterev assumed a host of roles, from leading expert in the neuroscientific underpinnings of child development to founder of innovative child‐study research centers to chairperson at major mobilizing events. Bekhterev was a busy man who routinely delegated research to others and he was most in his element as overall authority and patron, entrepreneur and leader, guide and overseer. Certain aspects of Bekhterev's involvement in child science could be seen as opportunistic—a matter of tapping into an area of topical scientific, social, and state interest, while pressing the right buttons to attract funding for his scientific empire. At the same time, Bekhterev was effective in using his high status and his growing network of laboratories, clinics, institutes, and journals to support large numbers of researchers engaged in the study of normal and pathological childhood.

What this article has been most interested in was the role that the very particular research program that Bekhterev in the late 1890s–1900s called “objective psychology,” and which he then, by 1917, renamed “reflexology,” played within Russian child science. I have argued that “objective psychology”/“reflexology” can in this context be productively analyzed as a scientific/intellectual movement or SIM—a framework for the transformation of a preexisting scientific landscape, which assumed not just an “innovative” research agenda, but also the coordinated mobilization of participants and resources, and the development of particular rhetorical tactics to support this. While “objective psychology”/“reflexology” was a SIM with ambitions to transform the human sciences in the widest sense, this article focused principally on its role in child science.

Bekhterev's most active participation in child science took place in the 1900s, which was also when he wrote his key work on “objective psychology” and when he established the Pedology Institute in St. Petersburg. “Objective psychology” and then “reflexology” acquired, for a while, visible presence within the Russian/Soviet human sciences, thanks to Bekhterev's charisma, activism, and organizational abilities. However, this program was, never hugely successful within the narrower field of child science; and this was so irrespective of Bekhterev's undeniable prominence and authority within Russia's child science network.

One major reason for this was that “objective psychology”/“reflexology” failed to adequately relate to and impact upon the strategic area of school education and the teaching profession as a vital target constituency. Although Bekhterev and his followers were conscious of this problem and did their best to appeal to educators, their program was unable to compete with rivals, such as A. P. Nechaev's “experimental pedagogy” in the prerevolutionary era or the host of new programs that arose within the “pedology” bandwagon in the early Soviet Union. The failure that was “pedagogical reflexology” and the niche subarea that “developmental reflexology” ended up as are testimonies to this.

The other reason for this lack of success is that Bekhterev fostered the study of child development mostly as a subordinate substructure within a larger edifice of the “psychoneurological sciences.” In this context, the developing child was of interest primarily as a “model organism” for the innovative reconceptualization of the emergence of higher mental functions. Indeed, as this article has argued, the infant and the toddler assumed a pivotal position in Bekhterev's efforts to ground in specific phenomena and in concrete research practices *a new ontology of the psychological*, which he simultaneously articulated in his abstract schema of “the reflex,” and which lay at the core of his program for transforming psychology as a discipline. While for most other players in Russian child science at this time, with A. P. Nechaev as the prime example, an *already transformed* scientific psychology was to be *built into* a newly emerging science of child development and socialization, for Bekhterev it was the other way around—studying child development made sense only as a function of his ambition to transform “the psychological” as such.

## Footnotes

1 On the rivalry between Pavlov and Bekhterev see Todes (2014, pp. 319–336). If Pavlov was a “manager” in science (Todes, 2002), then Bekhterev can best be described as an “entrepreneur.”

2 For accounts of Bekhterev's life see Guberman (1977), Nikiforov (1986), Brushlinskii and Kol'tsova (1997), Nikiforov, Amirov, and Mukhamedzianov (2007), and Akimenko (2007).

3 Watson found inspiration in the 1913 French translation of Bekhterev's *La psychologie objective* (Lecas, 2011).

4 For exceptions see Munilov (1968), Valsiner (1988, pp. 52–60), Martsinkovskaia (1990), and Balashov (2012, pp. 53–60, 73–78).

5 For a historical epistemology of “objectivity” see Daston and Galison (2007). This study focuses on the distinction between “mechanical objectivity” (the repression of the intervention of the authorial self in the recording of reality, e.g., through the use of photography) and “structural objectivity” (the reconstruction of the inner structures of reality, through logical and mathematical modeling).

6 According to Daston and Galison (2007, p. 261), both structural and mechanical objectivity are about epistemology, *not* ontology.

7 There were also other ways through which Bekhterev sought to do this. Key to much of his theorizing was the neovitalist concept of “energy” that (rhetorically and conceptually) shuttled across the physical, the neurological, the psychic and the social (Valsiner, 1988, p. 56; Valsiner, 2001).

8 On PNI, see Akimenko and Shereshevskii (1999–2002). It was anticipated that PNI would have a crèche and nursery (Dobrotvorskii, 1908).

9 The initial sum of 52,000 rubles was donated by V. T. Zimin, a philanthropist from Siberia with interest in education (Uchrezhdenie, 1907; Upravlenie, 1908; Protokoly, 1911). Zimin was actively involved in the setting up of PI in the early years. He had, in fact, independently come up with the idea of a boarding‐school nursery grounded in science and had sought partners among child science authorities, including Nechaev, but eventually partnered up with Bekhterev and remained on the Institute's board until the end of his life (Arkatov, 1908; Usovershenstvovanie, 1908; Tsentral'nyi Gosudarastvennyi Istoricheskii Arkhiv [hereafter TsGIA] f. 2265 op. 1 d. 933 ll. 1–76).

10 This was made possible through a grant from the tsarist Ministry of Public Education (TsGIA f. 2265 op. 1 d. 931 ll. 1–2; TSGIA f. 2265 op. 1 d. 926 ll. 1‐7; TsGIA f. 2265 op. 1 d.927 ll. 1‐5).

11 On the difficulties faced in the early days of PI see TsGIA f. 2265 op. 1 d. 933 ll. 1‐76, esp. ll. 55–76.

12 TsGIA f. 2265 op. 1 d. 933, ll. 55–76.

13 On the teaching of child psychology at PNI based on the work of PI see TsGIA f. 2265 op. 1 d. 929 ll. 1‐6ob, esp. ll. 3‐6ob, and also Povarnin (1913).

14 Initially, Bekhterev thought that film, rather than photography, would be the preferred technology and he was even somewhat dismissive of the latter (Bekhterev, 1908a). The use of a phonograph for the recording of sounds and speech was also envisaged.

15 For examples of research carried out at PI under the banner of “objective psychology,” including the study of respiration patterns, ability to distinguish colors, motor and emotive reactions, and children's drawings, see: Kostylev (1908); Vladychko (1909); Brazhas (1911, 1913); Pavlovskaia (1911); Kiterman (1912); Trudy (1914, pp. 266–349) and Bodyreva (1913, 1916).

16 This journal ran from 1904 to 1919 with a break in 1915. It was initially called *Herald of Psychology, Criminal Anthropology and Hypnosis* (*Vestnik psikhologii, kriminal'noi antropologii i gipnoza*), with the last part of the title changing from *Hypnosis* to *Pedology* in 1912. It became the official journal of PNI in 1907. In the 1920s, Bekhterev's key journal in child science was *Questions of the Study and Education of Personality* (*Voprosy izucheniia i vospitaniia lichnosti*), which came out between 1920 and 1932, with a break in 1923 to 1925.

17 Those participating in child science at PNI developed their own innovative methodologies, the most prominent being A. F. Lazurskii's “natural experiment”—a form of “objective psychology” on the boundary with “experimental pedagogy” (Byford 2014, pp. 30–31).

18 Bekhterev's paper at this conference was, however, on “suggestion” (*vnushenie*), based on his work in hypnosis (Mal'tseva, 1912).

19 This sometimes entailed “hijacking” their work without approval (see TsGIA f. 2265 op. 1 d. 933 ll. 1‐76)!

20 On the Pavlov‐Bekhterev clash see Todes (2014, pp. 319–336). On the differentiation between the two reflexologies see Lecas (2011).

21 The extent to which “the reflex” was for Bekhterev *both* a physiological phenomenon *and much more* is evident from the metaphorical language he used to describe it. He famously called the reflex a “life atom” (*zhiznennyi atom*), the idea of “life” being expandable beyond the narrowly physiological (Brushlinskii & Kol'tsova, 1997).

22 I here borrow and adapt the notion of “bandwagon” from Fujimura (1988).

23 “Experimental pedagogy” continued to feature as an approach within the child science bandwagon, but soon dissolved within the wider framework of “pedology.” Nechaev remained active in child science, taking part in various research commissions and training institutions, but he no longer positioned himself as a leader of a movement and his research shifted increasingly to labor psychology.

24 Bekhterev subdivided reflexology into applied substrands. Related to “pedagogical reflexology” was also the “reflexology of labor” (*refleksologiia truda*).

25 Osipova was one of the first graduates from the Women's Medical Institute (Drapkina, 1976). She worked in Bekhterev's St. Petersburg clinic (1902–1906) and at Kazan’ University (1906–1916). She returned to Petrograd to head the psychology laboratory at the Central Pedagogical Museum. From 1920, she was employed at the Brain Institute. Her laboratory there was referred to as “laboratoriia vozrastnoi refleksologii.” Both the terms *geneticheskaia* and *vozrastnaia* are translatable as “developmental”; the former entailed “early development,” while the latter referred to subsequent “growing up.” For a positive presentation of this laboratory see Osipova (1926). For a critique see Artemov (1929).

26 This was contemporaneous with Howard Liddell's conditioning experiments at his Behavioral Farm Laboratory (1924–1926), which used electric shocks under the influence of Soviet research (Lecas, 2011, p. 118).

27 It was Pavlov's student and associate, N. I. Krasnogorskii, who pioneered the application of Pavlovian experiments to children, focusing on the swallowing and salivation reflexes, publishing his results in 1907–1908 (Todes, 2014, pp. 312–13). Bekhterev (1912, pp. 4–5) was well aware of Krasnogorskii's work. Krasnogorskii also influenced the American psychologist Florence Mateer, whose book was translated into Russian in 1926 (Artemov, 1929, p. 37). Vsevolod Pudovkin's film *The Mechanics of the Brain* (1926), which popularized Pavlov's work, included demonstrations of Pavlovian experiments on children.

28 Not long before he died, Bekhterev featured in the line‐up of contributors to the newspaper *Pravda*’s bi‐weekly supplement *Revolution and Culture* (*Revoliutsiia i kul'tura*, November 15, 1927), alongside key Bolsheviks that Stalin was to eliminate or side‐line in the years to follow—namely, Deborin, Bukharin, Lunacharskii, and Krupskaia (Bekhterev, 1927).
